# Prevalence, Predictors, and Awareness of Coffee Consumption and Its Trend among Saudi Female Students

**DOI:** 10.3390/ijerph17197020

**Published:** 2020-09-25

**Authors:** Hanan A. Alfawaz, Nasiruddin Khan, Sobhy M. Yakout, Malak N. K. Khattak, Amani A. Alsaikhan, Areej A. Almousa, Taghreed A. Alsuwailem, Taghreed M. Almjlad, Nada A. Alamri, Sahar G. Alshammari, Nasser M. Al-Daghri

**Affiliations:** 1 Department of Food Science & Nutrition, College of Food Science & Agriculture, King Saud University, Riyadh 11451, Saudi Arabia; amanias1919@gmail.com (A.A.A.); areejalmousa55@gmail.com (A.A.A.); t.suwailem@gmail.com (T.A.A.); totaalmjlad@gmail.com (T.M.A.); nada.alamri1992@gmail.com (N.A.A.); sahar_gharbi@hotmail.com (S.G.A.); 2 Department of Biochemistry, College of Science, Chair for Biomarkers of Chronic Diseases, King Saud University, Riyadh 11451, Saudi Arabia.; syakout@KSU.EDU.SA (S.M.Y.) malaknawaz@yahoo.com (M.N.K.K.); aldaghri2011@gmail.com (N.M.A.-D.); 3 Department of Food Science and Human Nutrition, College of Applied and Health Sciences, A’ Sharqiyah University, Ibra 400, Sultanate of Oman; knasiruddin@asu.edu.om

**Keywords:** coffee, BMI, health awareness, academic performance, female students, Saudi Arabia

## Abstract

This study aimed to investigate the prevalence, trends, and predictors of coffee consumption among Saudi female students and its association with anthropometric and demographic variables. A survey-based study using a face-to-face interview was designed, and 930 (aged 21.5 ± 2.1 years) apparently healthy female students from different departments of King Saud University participated. The prevalence of coffee consumption was significantly higher (88.2%, *p* < 0.03) in the central Riyadh region. Coffee consumers had significantly higher prevalence of being overweight than non-consumers (*p* = 0.02). The frequency of coffee consumption was significantly higher (*p* < 0.02) in students who were single and belonged to families with a moderate income level. Coffee consumption was significantly higher among first-year students with a high-scale grade point average (GPA) (*p* < 0.001 and *p* = 0.03, respectively). Increased coffee consumption during exam and stress conditions was associated with unhealthy dietary habits such as using more sugar and spices. The prevalence of coffee consumption was high among Saudi females. High body mass index (BMI) and increased family income level were strong determinants for coffee consumption. Continued nutritional education and awareness about the potential positive and negative health effects of coffee consumption and the importance of food label use should be provided to younger generations in order to correct the wrong perceptions.

## 1. Introduction

Coffee is one of the most widely consumed beverages in the world. It is a complex chemical mixture containing hundreds of biologically active compounds including carbohydrates, lipids, nitrogenous compounds, vitamins, minerals, alkaloids, and phenolic compounds [1]. Caffeine, a substance that belongs to a group of compounds called methylxanthines, is one of the compounds with known biological activity. According to preparation techniques, the content of caffeine varies widely in different foods and beverages [2]. Generally, caffeine is responsible for the beneficial effects of coffee, but studies also suggest the role of other compounds in the health effects of coffee consumption [3].

Coffee has received a great deal of attention due to the high global prevalence of its consumption, and it is estimated to be increasing gradually in Asia, Africa, Europe, and North America [4]. The daily average caffeine consumption in United States (US) adults is estimated to be 180–190 mg of caffeine, equivalent to 2–3 cups of coffee [5]. Caffeine has both harmful and favorable health effects on the human body, such as anxiety, insomnia, tachycardia, irritability, and other cardiovascular disease (CVD) [6]. The health effects of caffeine depend largely on the dosage used, and it has been established that moderate consumption of coffee is associated with favorable health effects and behaviors [7]. On the basis of current research, the recommended portion for favorable and safe health effects of caffeine ranges between 300 and 400 mg/day of caffeine which is equivalent to 3–4 cups/day [8,9]. This dose is even less in the case of pregnant and lactating mothers [10]. The consumption of coffee is often associated with unhealthy eating behaviors leading to adverse health effects [11]. It has been reported that university students might be at a particularly high risk of negative effects due to the excessive intake of caffeine [5]. 

There were several studies presented from various regions in Saudi Arabia regarding caffeine and caffeinated energy drink consumption in university students. However, only limited studies are available from this country regarding the consumption of coffee in female university students. A study performed in Saudi Arabian dental students (*N* = 200, 98 females (20–25-year-olds), 102 males) showed higher consumption of caffeine in males than females. [12]. Another study performed by Ahmed and colleagues in young Saudi females (112 Saudi nonpregnant young women, age of 26 ± 1.85 years) observed a negative association between knowledge about caffeine and attitude toward use of caffeine during pregnancy [13]. Lack of awareness about caffeinated drink ingredients and unreliable resources as major sources of knowledge about their health effects were reported among Saudi adolescents and adults (*N* = 1062 subjects (867 males and 195 females) aged 18–60 years) [14]. 

Although the above-mentioned studies observed the relationship of caffeine consumption with many factors, including sociodemographic and behavioral [12,13,14], the results reported were biased due to either a low response rate or a small number of participants, specifically regarding females. Moreover, there is no study in Saudi Arabia observing the prevalence, determinants, use pattern, and awareness of coffee consumption in young female students. To fill this gap, the present study aimed to report the prevalence and observe the pattern of coffee consumption and its association with factors including obesity (body mass index (BMI), waist circumference (WC), waist-to-hip ratio (WHR)), sociocultural aspects, health behaviors, symptoms, and awareness. Moreover, to our knowledge, the present study is one of the first of its kind including such a large number of young female participants.

## 2. Materials and Methods 

### 2.1. Study Design 

In this cross-sectional study, 930 female students (which represents 5% of the King Saud University (KSU) population) were recruited from different colleges (Humanities, Health, and Science colleges) of KSU (Medicine, Dentistry, Nursing, Medical, Science, Computer and Information, Pharmacy, Arts, Education, Languages, Business Administration, and Law and Political Science) in Riyadh, Saudi Arabia. A pilot study of 50 students was performed to confirm the reliability and validity of the questionnaire. Content and face validity were done to clarify all questions. A written informed consent form was obtained from each participant. The questionnaire was then reviewed by experts in the related fields. Moreover, external reviewers provided their feedback and opinion in developing/improving the questionnaire to ensure reliability of the test. Expert feedback and suggestions were incorporated in the final questionnaire. Furthermore, Cronbach’s α, an estimate of reliability, was 84%, as measured for the whole questionnaire. A face-to-face questionnaire was completed. The questionnaire was divided into various parts including (1) anthropometric measurements (BMI values were used to classify participants as follows: normal (<25 kg/m^2^), overweight (25–29.9 kg/m^2^), and obese (≥30 kg/m^2^)), sociodemographic measures, and family income (less than 3000 Saudi Arabia riyals (SAR) was considered low income, between 3000 and 9000 SAR was considered average income, 10,000–27,000 SAR was considered moderate income, and more than 27,000 SAR was considered high income); (2) academic information such as specialty of study and grade point average GPA; (3) health information; (4) coffee consumption, duration, type of coffee, size of cup (different samples of cup sizes were used to make sure that interviewers could give an accurate size), and caffeine content; (5) food habits and reading food labels related to coffee consumption; (6) health-related knowledge associated with coffee consumption; (7) symptoms related to coffee consumption (self-reported by participants and not measured). This study was approved by the Ethics Committee for Scientific Research and Post Graduate Studies at the College of Science, King Saud University, Saudi Arabia (reference# KSU-HE-20-322).

### 2.2. Statistical Analysis

Data were analyzed using the Statistical Package for Social Sciences (SPSS) 22.0 (SPSS Inc., Chicago, IL, USA). All categorical variables were presented as frequencies and percentages (%), and their associations were evaluated using chi square and Fisher’s exact test. Continuous data were presented as means ± standard deviation (SD). All continuous variables were checked for normality using the Kolmogorov–Smirnov test. An independent *t*-test was used to check the mean difference for continuous variables between coffee consumers and non-consumers in terms of age, BMI, WHR, etc. All *p*-values were two-tailed; a *p*-value <0.05 was considered statistically significant. 

To examine the predictors of coffee consumption, the binary (crude data) and multivariate adjusted odds ratios (ORs) with 95% confidence intervals (95% CIs) were calculated using logistic regression models. All models were mutually adjusted for all potential confounders. The multivariate-adjusted models included potential determinants of coffee consumption such as BMI, family income level, number of family members, region, GPA, academic year, and medical history. A *p*-value <0.05 was considered statistically significant. Nagelkerke *R*^2^ was used to explain the variation in dependent variables and the Hosmer–Lemeshow goodness-of-fit test was also performed to suggest if the model was a good fit to the data (*p* ≥ 0.05).

## 3. Results

A total of 930 respondents, aged 21.5 ± 2.1 years, completed the questionnaire. The percentage of participants consuming coffee was higher (*n* = 820, 88.2%) than non-consumers (*n* = 110, 11.8%). The frequency of coffee consumption on a daily and weekly basis was 61.1% and 30.6%, respectively, among students (Table 1). 

### 3.1. Sociodemographic Characteristics and Anthropometrics

Table 2 represents the anthropometric and sociodemographic characteristics of our study population. Female students consuming coffee were observed to have a significantly higher BMI than non-consumers (*p* = 0.01). A significantly higher frequency of students consuming coffee fell into the overweight category than non-consumers (21.5% vs. 18%, *p* = 0.02). No significant difference was observed in the frequency of coffee consumption among students from different departments (Humanities, Health, and Science colleges). The frequency of coffee consumption was significantly higher (*N* = 770 (93.9%); *p* = 0.018) in single than in married participants (*N* = 90 (87%)). Although the pattern was not entirely uniform, the results demonstrated a significantly higher frequency of coffee consumption among students with increased family income level (from low to moderate: 3000–21,000 SAR, *p* = 0.038). In addition, the frequency of coffee consumption was significantly higher (*p* = 0.027) in students who were originally from the central region as compared to other regions of Saudi Arabia. Increased coffee consumption was observed among students with high (excellent and very good) compared to low GPA scores (*p* < 0.001). A significantly lower percentage (*p* = 0.03) of coffee consumption was reported in final-year students (fifth and sixth) compared to first-year students (Table 2). No significant statistical difference was observed among coffee consumers vs. non-consumers related to their medical history. 

### 3.2. Coffee Consumption Trend

Among different types of coffee consumed, the prevalence of Arabic coffee consumption (*N* = 641 (68.9%)) along with caffeine (*N* = 606 (94.6%)) was observed to be highest with an average consumption of 4.8 ± 2.8 cups per day. The frequency of cup size used was mostly small (*N* = 387 (60.3%)). Following Arabic coffee, cappuccino and mocha (both with caffeine) were other coffee types consumed among students with a prevalence of 37.1% and 30.9%, respectively. The prevalence of latte and Turkish coffee consumption was about 21.8% and 17.3%, respectively (Table 3). 

### 3.3. Health Awareness about Coffee Consumption

Table 4 shows the participants’ health-related knowledge associated with coffee consumption. On the basis of their responses, the awareness of coffee consumers was significantly high and they were well informed compared to non-consumers on associations such as the following: coffee reduces tiredness (*p* = 0.005), excessive use can increase pathological conditions (*p* = 0.002), the need for moderation during pregnancy (*p* = 0.04), it can improve intellectual abilities (*p* < 0.001), it can increase anxiety (*p* = 0.02), and some pain relievers contain caffeine (*p* = 0.05). In contrast, more non-consumers than consumers (74.6% and 62.6%, *p* = 0.01) were aware that coffee consumption causes insomnia. A higher proportion (41.7%, almost significant, *p* = 0.07) of consumers believed that drinking coffee increases blood pressure than non-consumers (35.6%). In addition, although nonsignificant, the majority of consumers and non-consumers were aware that coffee acts as a stimulant (92.7% and 92.1%, respectively) and, if taken in excess, may increase heart rate (68.3% and 62.7%, respectively).

### 3.4. Determinants of Coffee Consumption

The independent determinants of coffee consumption zre shown in Table 5. The results of multivariate-adjusted odds ratios (ORs) demonstrated high BMI and high income level as independent predictors of coffee consumption among female students. The odds of coffee consumption were 4.42-fold (95% CI: 1.15–17.1) higher in obese students than in those with normal BMI. Moreover, the probability of coffee consumption was lowest (OR: 0.15, 95% CI: 0.02–0.99) in students with low family income level (<3000 SAR). The unadjusted odds ratios showed the high probability (OR: 3.37 (95% CI:1.25–9.02)) of coffee consumption by those with a moderate number of family members (6–8). However, this association was lost when adjusted for confounding variables.

### 3.5. Food Habits and Reading Food Labels Associated with Coffee Consumption 

The food habits associated with coffee consumption are presented in Table 6. Among coffee consumers (*N* = 820), the frequency of adding sugar (42.9%) and spices such as saffron and cardamom (71.9%) was high. However, most of them did not habitually add artificial flavors (44.4%), sauces (54.7%), and cream (68.5%) on a regular basis. The majority of coffee consumers (70.9%) responded negatively when asked about drinking coffee immediately after meals, and around 42.2% of them habitually drank coffee before breakfast. More importantly, the majority of coffee consumers (64.6%) increased their coffee intake during exams and stressful situations.

The frequency of coffee consumers showing interest in reading the food label for coffee products was very low (13.3%), and only 24.4% of the consumers were able to understand the contents of the food label. Moreover, the frequency of consumers highlighting the positive role of food labels in purchasing behavior was low (21.1%), while the proportion of coffee consumers interested in knowing about the ingredients of coffee from coffee shops was only 22.9%.

### 3.6. Health Symptoms Related to Coffee Consumption 

**Figure 1:**
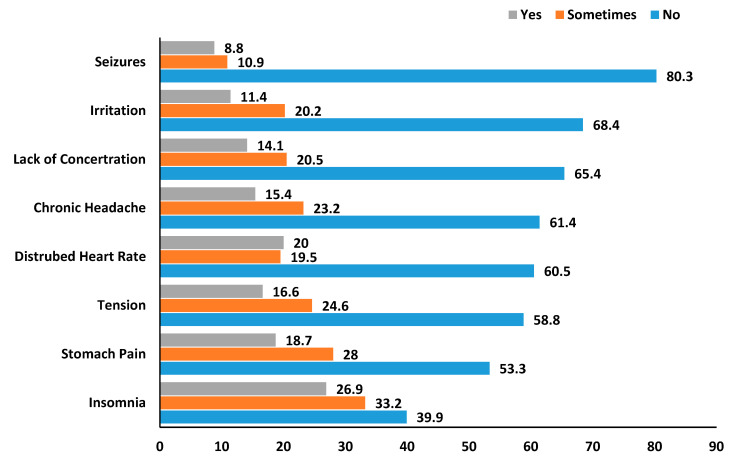
The frequency of symptoms related to coffee consumption.

Figure 1 summarizes the symptoms related to coffee consumption. Among those who consumed coffee, only few students reported insomnia (*p* = 0.005), stomach pain (*p* = 0.021), disturbed heart rate (*p* = 0.030), and tension (*p* = 0.024).

## 4. Discussion

Over the past few decades, the tradition of coffee consumption has grown in popularity in Middle Eastern countries, including Saudi Arabia. The present study highlighted a high prevalence (88.2%) and trends of coffee consumption among female university students. Moreover, the study indicated strong predictors of coffee consumption to be high BMI and high family income level. 

The consumption of caffeine depends on age and, therefore, main dietary caffeine sources may differ as age advances [15]. The reports from The National Health and Nutrition Examination Survey NHANES showed positive linear trends between age and total caffeine consumption until 45–55 years of age [5]. Around 70% of United States (US) children and adolescents (aged <22 years) consume some form of caffeine on any given day, and approximately 89% of adults consume caffeinated beverages on a daily basis [16,17]. Moreover, the prevalence of caffeine consumption among Hungarian males and females is quite high (91.5% and 93.2%, respectively) [18]. A study among students (aged 18–24 years) at the University of Sarajevo, Bosnia and Herzegovina, demonstrated a prevalence of 71.2 % (*N* = 673) coffee consumption [19]. The prevalence of caffeine intake per day among students from six different universities in Bahrain was high (98%), with coffee as the main source of caffeine intake (76%) [20]. A similar study reported 72.3% coffee intake among Brazilian students with a higher prevalence among females (68.3%) [21]. Moreover, a study performed in United Arab Emirates (UAE) university students (male 30.1% and female 69.9%) showed a high prevalence (85.1%) of caffeinated energy drink consumption [22]. A high prevalence (86%) of caffeinated drink consumption was observed among students at Zayed University, UAE, with coffee as the major contributor of caffeine intake [23].

The prevalence of coffee consumption in our present study is considerably high (88.2%) as compared to Bahrain (76%), Brazil (68.3%), US (70%), and Bosnia and Herzegovina adolescents (71.2%), and it is nearly equal to UAE university students (86%).

The possible reasons for this high consumption can be mainly attributed to the culture and traditions of this country. Like other Middle Eastern countries, the consumption of Arabic coffee is a social norm and an essential part of hospitality, which is highly practiced for every occasion and event [24]. Saudi families drink Arabic coffee 1–2 times daily and most of them drink coffee after evening prayer (Maghreb) around 6:00–7:00 p.m. [25]. Moreover, in Arab and other Muslim countries, caffeinated beverages are the only popular drink products which are quite common and easily available to university students. 

The consumption of coffee and its effect on body weight and body fat distribution is still controversial. Coffee contains several biologically active compounds that result in beneficial effects on obesity and other interrelated abnormalities. However, there are studies reporting direct [26], inverse [27], and neutral relationships between coffee consumption and obesity [28]. Variations in coffee composition and consumption pattern (such as coffee type and amount of intake) are major factors for the inconsistent effect of coffee on human health. Moreover, as abnormal lipid metabolism is common in conditions such as obesity, diabetes, and metabolic syndrome, the effects of coffee bioactivity on lipid metabolism are suggested as underlying mechanisms of its positive health effects [9]. The health effects of Arabic coffee consumption are still controversial with a lack of scientific evidence about the benefits and/or risk factors. The consumption of Arabic coffee in healthy individuals was shown to result in high total serum cholesterol level as compared to non-consumers, especially in females [29]. A study performed in healthy women consuming Arabic coffee with added spices like cardamom demonstrated a neutral effect on blood pressure and biomarkers of inflammation (e.g., C-reactive protein), but resulted in an increased level of low-density lipoprotein cholesterol and total cholesterol [30]. In addition, Arabic coffee consumption in healthy individuals modestly increased the plasma glucose response of dates compared to that of dates consumed with water, probably due to the presence of caffeine, which could impair glucose tolerance and decrease insulin sensitivity [31]. A recent study of Taibah university students and employees (18–45 years of age) demonstrated a high level of Arabic coffee consumption and its positive association with obesity (28% overweight and 33% obese), indicating a health warning against its excessive intake [32]. Our present study supports the above findings [26,32], showing a direct association between coffee consumption and BMI among female students.

The association of coffee consumption with marital status is still inconsistent with mixed data. It was reported that married women consume caffeinated beverages nearly twice as frequently as unmarried women [33]. However, a study performed at Zayed University, UAE, demonstrated a higher consumption of coffee in single than in married females [23], which is similar to the findings of our present study. In addition, it was postulated that marital status and having children could bring a difference in dietary consumption and other lifestyle behaviors. Studies suggests that married men and women have healthier dietary behaviors compared to unmarried men and women, and this could be another reason for the lower coffee consumption among married students [34,35]. 

A study from the Hail region in Saudi Arabia demonstrated the increased used of caffeinated energy drinks in self-dependent low-income participants compared to those with high income [14]. Similarly, a Korean study suggested a direct association between high household income level and the prevalence of coffee consumption [33]. Moreover, low-income adults were shown to consume less caffeine than higher-income adults [34]. The present study supports the above findings [36,37], demonstrating a significant increase in the prevalence of coffee consumption with an increase in family income level. The direct effect of income and coffee consumption was somewhat diminished in the high-income level since the majority of the participants belonged to the moderate range of family income (10,000–27,000 SAR). Our present study demonstrated a high proportion of coffee consumption (57.5%) within families having 6–8 members. As far as family size is concerned, reports suggested that the poverty–income ratio (PIR), an index calculated by dividing family income by a federal poverty threshold specific to family size, generally used as a proxy for socioeconomic status, was not associated with caffeine consumption [15].

There are reports on the consumption of caffeinated energy drinks from different regions of Saudi Arabia [38]; however, until recently, limited information was available about coffee consumption from different region of the capital city. Similar to a Korean study [36], our present study observed the highest consumption of coffee in students originally from the central Saudi Arabia region than any other part of the Kingdom of Saudi Arabia. 

Caffeine, being a central nervous system and metabolic stimulant, is capable of reducing fatigue and inducing alertness along with improving performance during sleep deprivation [39]. Considering its effect on performance, students generally take it to keep awake during study. The high prevalence of caffeine intake in German students exclusively for the purpose of cognitive enhancement is much higher than the prevalence rate of stimulants [40].

Coffee’s long-term effect on cognitive function remains unclear with studies suggesting both benefits and adverse effects [41].A protective effect of coffee use was reported against cognitive impairment or decline, showing stronger effects among females than males [42], but this study lacked a dose–response relationship. It was demonstrated that caffeine consumption resulted in higher levels of alertness, energy, concentration, and arousal level during lecture time as compared to placebo [43]. With respect to cognitive function and alertness, the results of our present study support the above findings [39,42,43], demonstrating higher academic performance among students consuming increased amounts of coffee. Moreover, in the present study, the majority of students generally got 6–8 hours of sleep, and this could have had a favorable effect on maintaining concentration during classroom lectures, leading to better academic performance. The freshman students in the present study showed a higher consumption of coffee as compared to final-year students. A possible explanation is that a freshman is more susceptible to stress due to their new course burden and study environment, leading to several coping methods, such as more coffee consumption.

In Saudi Arabian culture, the use of “qahwa” or Arabic coffee, as a hot beverage which is generally served in a small cup, is regular practice during many special occasions [24]. Among different types of coffee consumption, our present study demonstrated a higher use of Arabic coffee. The mean intake of different types of coffee consumption ranged from 2.0 ± 1.6 cups/day (mocha) to 3.1 ± 2.6 (Americano), which falls within the acceptable range of many dietary recommendations [8,9]. However, the mean intake of Arabic coffee was slightly high with 4.8 ± 2.8 cups/day due to social and cultural trends, as explained earlier.

The results of our study showed that awareness about the health benefits of coffee consumption among consumers was considerably higher than in non-consumers, but some misconceptions and doubts were also observed. Studies demonstrated that only 16% of US consumers know about coffee’s health benefits, while 49% of European consumers believe that coffee may cause health problems [44,45]. Hence, positive health impacts provided by coffee may incline consumers toward consumption, while negative heath impacts are key factors for not drinking coffee. An increased consumption of caffeine or caffeinated energy drinks was reported during stress conditions such as exams [46]. In the present study, the majority of coffee consumers were well aware that coffee is a stimulant (92.7%), reduces tiredness, and causes insomnia [47]. It is well known that high doses of coffee can lead to negative health effects in users, such as tachycardia, palpitations, and anxiety [48]. Among consumers, only 39.1% responded correctly that excess coffee intake is associated with increased anxiety, while the majority (68.3%) of them were well aware about the association of excess coffee dose with increased heart rate and conductivity. More than half of the coffee consumers (55.7%) were aware that there is a need to moderate coffee intake during pregnancy, but only 41.7% responded positively that acute, moderate intake of caffeine (1–3 mg/kg) in adults and children increases blood pressure [49]. About 49.3% of coffee consumers were aware that low doses of caffeine are present as an adjuvant in many over-the-counter analgesics such as cold and allergy tablets and headache medications, which may prove useful without any serious adverse health effects [50]. Although students consuming coffee have information that it improves intellectual abilities, as discussed earlier, this topic is still inconsistent and needs further studies in the future. 

An increase in academic load is often related to stress [46], leading to increased coffee consumption with negative consequences such as unhealthy behavior, bad lifestyle, and poor dietary habits [5,11]. The consumption of caffeinated beverages, especially coffee and soft drinks, increases among students during high-stress conditions such as exams. A study from Puerto Rico demonstrated a high consumption of coffee among students used to stay awake during exams [51]. Stressed female students in Kuwait were reported to consume unhealthy snacks with high sugar and fat content along with high amounts of beverages [52]. A similar study among university students in Kuwait demonstrated unhealthy dietary habits such as adding sugar while consuming tea or coffee [53]. Similarly, our present findings demonstrate a high consumption of coffee among majority of our participants during exam and stress conditions, associated with unhealthy dietary habits such as adding sugar and spices.

Drinking qahwa with dates or other sweets is an Arabian staple. Unlike typical European black coffee, qahwa is prepared with two main ingredients: blond coffee beans and cardamom. Optional spices include saffron, ginger, cloves, etc. [32]. According to the cultural method of preparing Arabic coffee, the participants of our present study obviously used more spices like cardamom and saffron during its preparation and consumption.

The temporal distribution pattern of caffeine consumption among the US population (except for adolescents) occurs predominantly in the morning, driven primarily by coffee. However, adolescents distribute their caffeine consumption throughout the day [54]. Similarly, about 42.2% of our coffee consumers reported habitual consumption before breakfast, while the rest distributed it throughout other times of the day.

Nutritional information on food labels affects consumers purchasing attitude and behavior, prompting healthy food choices and serving as a good rationale to buy them [55]. The significant determinants for frequent label use include health reasons, specific nutrition information, weight control, and knowledge, while determinants of infrequent label use include purchasing food regardless of nutrient content, time constraints, and a “do not care” attitude [56]. In the present study, the majority of coffee consumers were not very concerned with reading food labels, showing their ignorance toward the positive effects of food label use during coffee purchase and product selection. In addition, the consumers did not have much of an understanding about the contents of the food label.

The majority of participants responded negatively with respect to any frequent side effects and symptoms associated with coffee consumption. However, these symptoms (insomnia, stomach pain, disturbed heart rate, and tension) were present in some students but not long-lasting. A possible reason for this effect may be the excessive consumption of caffeine along with artificial flavors or spices that could have a stimulant impact on the body. Moreover, increased coffee consumption can lead toward such side effects, mostly in females [57].

Few limitations should be considered while interpreting the results of this study. First, the sample size is high but does not represent the overall adult female population of Saudi Arabia. Second, due to its cross-sectional nature, no plausible causation can be obtained from our present study. Although we adjusted the odds ratios in our logistic regression, the potential for unmeasured and residual confounding factors may still exist. The questionnaires did not include reference to the size of a cup of coffee, which could have led to misclassification of mean cup intake. Moreover, the information about variables such as coffee consumption and academic performance could have led to recall bias.

Despite these limitations, our study has numerous strengths. To our knowledge, the present study is a novel contribution to the literature in observing the simultaneous cross-sectional association between coffee consumption and various sociodemographic variables, health awareness, associated food habits, health symptoms, and consumption determinants. The trends of coffee consumption were examined specifically for various types of coffee consumed among Saudi students. Moreover, the present study used a pretested questionnaire for a representative sample.

## 5. Conclusions

According to the present observations, the cultural trend of consuming Arabic coffee in Saudi females is increasing significantly, confined mostly in the central Saudi Arabia region. The strong predictors of coffee consumption among female students were high BMI and high family income level. Prolonged study during stress conditions, such as exams, and better academic performance were some of the major rationales for the high intake of coffee among first-year students. In spite of having sufficient health awareness, this study observed a lack of recent information and doubts regarding the health effects of coffee and label use among a considerable proportion of consumers. To overcome this problem, awareness programs regarding the adverse effects of high coffee consumption and nutritional education programs encouraging the importance of reading food labels and their use in purchasing should also be raised in the future. Considering our results as a baseline and adding more information such as the motives and profile of coffee consumers, additional research will be needed to continue to monitor these trends among Saudi adolescents and adults.

## Figures and Tables

**Table 1:** Demographic characteristics of participants.

Parameters	Frequency (%)
*N*	930
Drink coffee (Yes)	820 (88.2)
**Frequency of coffee intake**	
Daily	501 (61.1)
Weekly	250 (30.6)
Every 2 weeks	24 (2.8)
Every 3 weeks	25 (3.0)
Every month	20 (2.5)
**Departments**	
Medical	42 (4.5)
Dentistry	23 (2.5)
Nursing	33 (3.5)
Applied Medical	64 (6.9)
Science	277 (29.7)
Computer and Information	131 (14.1)
Pharmacy	12 (1.3)
Arts	57 (6.1)
Education	88 (9.5)
Languages	70 (7.5)
Business Administration	78 (8.4)
Law and Political Science	55 (6.0)

**Table 2:** Differences in demographic and anthropometric characteristics of coffee consumers vs. non-consumers.

Parameters	All	Coffee Consumption		*p*-Value
		Yes	No	
*N*	930	820	110	
**Age (years)**	21.5 ± 2.1	21.5 ± 2.1	21.7 ± 2.0	0.57
**BMI (kg/m^2^)**				
Normal (<25 kg/m^2^)	641 (68.9)	554 (67.6)	87 (79.0)	
Overweight (25–29.9 kg/m^2^)	199 (21.4)	179 (21.5)	20 (18.0)	**0.02**
Obese (≥30 kg/m^2^)	90 (9.7)	87 (10.9)	3 (3.0)	
**Colleges**				
Science	592 (63.7)	524 (63.9)	68 (62.0)	0.39
Arts	338 (36.3)	296 (36.1)	42 (38.0)	
**Marital Status**				
Single	860 (92.5)	770 (93.9)	90 (87.0)	**0.018**
Married	70 (7.5)	50 (6.1)	20 (13.0)	
**Family Income (SAR)**				
<3000	9 (1.0)	7 (0.8)	2 (1.8)	
3000–9000	175 (18.8)	165 (20.1)	10 (9.1) *	
10,000–15,000	219 (23.5)	174 (21.2)	45 (40.9) *	**0.038**
16,000–21,000	272 (29.3)	245 (29.9)	27 (24.5)	
22,000–27,000	116(12.5)	98 (12.0)	18 (16.4)	
>27,000	139 (14.9)	131 (16.0)	8 (7.3)	
**No. of Family Members**				
2	24 (2.6)	18 (2.2)	6 (5.4)	
3–5	153 (16.5)	132 (16.0)	21 (19.1)	
6–8	525 (56.4)	472 (57.6)	53 (48.2)	
9–11	185 (19.9)	162 (19.8)	23 (20.9)	0.07
12–14	37 (4.0)	30 (3.7)	7 (6.4)	
>14	6 (0.6)	6 (0.7)	0 (0.0)	
**Sleep Hours**				
<3	10 (1.1)	7 (0.9)	3 (3.0)	
3–5	227 (24.4)	203 (24.8)	24 (22.2)	0.34
6–8	573 (61.6)	501 (61.1)	72 (64.6)	
9–12	100 (10.8)	90 (10.9)	10 (9.2)	
>12	20 (2.1)	19 (2.3)	1 (1.0)	
**Region**				
North Saudi Arabia	50 (5.4)	43 (5.2)	7 (6.4)	
South Saudi Arabia	170 (18.3)	138 (16.9)	32 (29.0) *	
East Saudi Arabia	36 (3.8)	29 (3.5)	7 (6.4)	**0.027**
West Saudi Arabia	93 (10.0)	81 (9.9)	12 (10.9)	
Central Saudi Arabia	581 (62.5)	529 (64.5)	52 (47.3) *	
**GPA**				
Excellent (4.51–5.0)	351 (37.7)	305 (37.2)	46 (41.8)	
Very Good (3.51–4.50)	473 (50.9)	422 (51.5)	51 (46.3) *	
Good (2.51–3.50)	98 (10.5)	92 (11.2)	6 (5.5)	**<0.001**
Acceptable (1.51–2.50)	2 (0.2)	1 (0.1)	1 (0.9)	
<1.51	6 (0.7)	0 (0.0)	6 (5.5) *	
**Academic Year**				
First year	132 (14.0)	114 (14.0)	18 (16.5)	
Second year	256 (27.9)	224 (27.3)	32 (28.6)	
Third year	190 (20.6)	177 (21.6)	13 (12.1) *	**0.03**
Fourth year	197 (21.0)	179 (21.7)	18 (16.5)	
Fifth year	71 (7.7)	60 (7.3)	11 (9.8)	
Sixth year	84 (8.8)	66 (8.1)	18 (16.5)	
**Medical History**				
T1DM	6 (0.7)	4 (0.6)	2 (2.1)	0.1
T2DM	0 (0.0)	0 (0.0)	0 (0.0)	0.15
Hypertension	12 (1.3)	12 (1.6)	0 (0.0)	0.22
Heart disease	21 (2.3)	21 (2.7)	0 (0.0)	0.11
Anemia	209 (22.3)	187 (22.8)	22 (19.8)	0.5
Convulsions or epilepsy	6 (0.6)	6 (0.7)	0 (0.0)	0.41
Asthma	76 (8.5)	62 (7.6)	14 (12.4)	0.11
Kidney disease	5 (0.5)	4 (0.4)	1 (1.1)	0.41
Pregnancy	7 (0.7)	5 (0.6)	2 (1.8)	0.57
Breastfeeding	4 (0.4)	4 (0.4)	0 (0.0)	0.6
Smoking (cigarettes)	11 (1.2)	9 (1.1)	2 (2.1)	0.42
Smoking (shisha)	9 (1.0)	7 (0.8)	2 (2.1)	0.25

BMI: Body Mass Index, SAR: Saudi Riyal, GPA: Grade Point Average, T1DM: Type 1 diabetes and T2DM: Type 2 diabetes. Superscript * represented *p*-value for subgroup (2*2) Chi-square value at 0.05 level of significance.

**Table 3:** Frequency of coffee consumption by type.

Coffee Type	Arabic	American	Mocha	Cappuccino	Latte	Turkish	Espresso	Frappuccino	Other
Coffee intake (yes)	641 (68.9)	192 (20.6)	287 (30.9)	345 (37.1)	203 (21.8)	161 (17.3)	91 (9.8)	111 (11.9)	61 (6.6)
Mean cup intake	4.8 ± 2.8	3.1 ± 2.6	2.0 ± 1.6	2.4 ± 1.9	2.6 ± 2.3	3.0 ± 3.0	2.4 ± 2.2	2.0 ± 2.0	2.9 ± 2.5
**Cup size**									
Small	387 (60.3)	89 (46.3)	128 (44.6)	141 (40.9)	86 (42.4)	148 (82.4)	75 (82.4)	51 (46.0)	20 (32.9)
Medium	129 (20.1)	75 (39.1)	128 (44.6)	181 (52.5)	106 (52.2)	13 (17.6)	16 (17.6)	50 (45.0)	34 (55.7)
Large	73 (11.4)	24 (12.5)	22 (7.7)	21 (6.0)	10 (4.9)	0 (0.0)	0 (0.0)	8 (7.2)	6 (9.8)
Extra large	26 (4.1)	4 (2.1)	4 (1.4)	2 (0.6)	1 (0.5)	0 (0.0)	0 (0.0)	2 (1.8)	1 (1.6)
Others	26 (4.1)	0 (0.0)	5 (1.7)		0 (0.0)	0 (0.0)	0 (0.0)	0 (0.0)	0 (0.0)
**Caffeine content**									
Decaffeinated	35 (5.4)	7 (3.8)	24 (8.2)	12 (3.5)	19 (9.1)	11 (6.5)	7 (7.6)	10 (9.3)	10 (16.4)
With caffeine	606 (94.6)	185 (96.2)	263 (91.8)	333 (96.5)	184 (90.9)	150 (93.5)	84 (92.4)	101 (90.7)	51 (83.6)

**Table 4:** Health awareness associated with coffee consumption.

Knowledge about Coffee Consumption	Coffee Consumers			Non-Consumers			
	Yes	No	Did Not Know	Yes	No	Did Not Know	*p*-Value
A stimulant	760 (92.7)	31 (3.8)	29 (3.5)	101 (92.0)	3 (3.2)	6 (4.8)	0.85
Reduces tiredness	417 (50.9)	244 (29.8)	159 (19.3)	37 (33.3)	54 (49.2)	19 (17.5)	0.005
Causes insomnia	513 (62.5)	197 (24.1)	110 (13.4)	82 (74.6)	9 (7.9)	19 (17.5)	0.01
In large doses leads to hallucination	228 (27.8)	262 (32.0)	330 (40.2)	37 (33.9)	32 (29.0)	41 (37.1)	0.6
In excess leads to pathological conditions	439 (53.5)	168 (20.5)	213 (26.0)	82 (74.6)	20 (18.6)	8 (6.8)	0.002
Needs moderation during pregnancy	457 (55.7)	95 (11.6)	268 (32.7)	56 (50.8)	4 (3.4)	50 (45.8)	0.04
Causes hypertension	343 (41.7)	121 (14.8)	357 (43.5)	39 (35.6)	7 (6.8)	63 (57.6)	0.07
Associated with weight loss	198 (24.2)	269 (32.8)	353 (43.0)	25 (22.0)	35 (32.2)	50 (45.8)	0.9
In excess increases heart rate	560 (68.3)	94 (11.5)	166 (20.2)	68 (62.7)	8 (6.8)	34 (30.5)	0.13
Improves intellectual abilities	341 (41.6)	202 (24.6)	277 (33.8)	27 (24.6)	62 (56.1)	21 (19.3)	<0.001
In excess increases anxiety	321 (39.1)	196 (23.9)	303 (37.0)	37 (33.9)	13 (11.9)	60 (54.2)	0.018
Associated with blood glucose control	161 (19.6)	165 (20.2)	494 (60.2)	15 (13.6)	28 (25.4)	67 (61.0)	0.41
Some pain relievers contain caffeine	404 (49.3)	61 (7.4)	355 (43.3)	37 (33.9)	8 (6.8)	65 (59.3)	0.05

Note: Data are presented as frequency (%); significant at *p* < 0.05.

**Table 5:** Logistic regression model determining independent predictors of coffee consumption in Saudi female students.

Parameters	Nagelkerke *R^2^*	Hosmer and Lemeshow Test	Crude		Multivariate-Adjusted	
			Odds Ratio (95% CI)	*p*-Value	Odd Ratio (95% CI)	*p*-Value
**Obesity**						
Normal (<25 kg/m^2^)			Ref		Ref	
Overweight (25–29.9 kg/m^2^)	0.019	1.00	1.40 (0.82–2.42)	0.22	1.77 (0.93–3.35)	0.08
Obese (≥30 kg/m^2^)			3.76 (1.16–12.25)	**0.028**	4.42 (1.15–17.1)	**0.031**
**Family Income (SAR)**						
<3000	0.062	1.00	0.18 (0.03–1.08)	0.06	0.15 (0.02–0.99)	**0.049**
3000–9000			0.98 (0.36–2.72)	0.97	1.06 (0.37–3.05)	0.91
10,000–15,000			0.24 (0.10–0.54)	**0.001**	0.23 (0.10–0.53)	**0.001**
16,000–21,000			0.56 (0.24–1.37)	0.21	0.61 (0.25–1.48)	0.27
22,000–27,000			0.35 (0.14–0.89)	**0.028**	0.34 (0.13–0.89)	**0.020**
>27,000			Ref		Ref	
**No. of Family Members**						
2			Ref		Ref	
3–5	0.021	1.00	2.35 (0.81–6.75)	0.11	2.17 (0.68–6.9)	0.18
6–8			3.37 (1.25–9.02)	**0.016**	2.82 (0.94-8.50)	0.07
9–11			2.58 (0.91–7.35)	0.07	2.21 (0.71–6.9)	0.17
12–14			1.45 (0.41–5.01)	0.56	1.33 (0.32–5.42)	0.69
>14			0.68 (0.31–1.35)	0.95	0.48 (0.03–0.68)	0.98
**Region**						
North Saudi Arabia			Ref		Ref	
South Saudi Arabia	0.024	1.00	0.69 (0.27–1.82)	0.46	0.61 (0.23–1.61)	0.32
East Saudi Arabia			0.68 (0.19–2.35)	0.55	0.55 (0.16–1.95)	0.36
West Saudi Arabia			1.05 (0.36–3.05)	0.93	0.83 (0.28–2.45)	0.73
Central Saudi Arabia			1.52 (0.62–3.79)	0.36	1.41 (0.56–3.54)	0.46
**GPA**						
Excellent (4.51–5.0)			Ref		Ref	
Very Good (3.51–4.50)	0.055	1.00	0.86 (0.54–1.37)	0.53	0.84 (0.52–1.37)	0.49
Good (2.51–3.50)			1.23 (0.55–2.78)	0.61	1.18 (0.51–2.73)	0.69
Acceptable (1.51–2.50)			0.12 (0.01–1.97)	0.134	0.12 (0.01–2.08)	0.15
<1.51			0.001 (0.00–0.001)	0.99	0.02 (0.001–0.04)	0.96
**Academic Year**						
First year			Ref		Ref	
Second year			1.13 (0.57–2.22)	0.73	1.03 (0.51–2.07)	0.93
Third year	0.028	1.00	2.11 (0.93–4.78)	0.07	2.23 (0.94–5.25)	0.07
Fourth year			1.56 (0.73–3.32)	0.25	1.71 (0.75–3.86)	0.2
Fifth year			0.87 (0.36–2.11)	0.75	0.95 (0.37–2.48)	0.91
Sixth year			0.58 (0.26–1.27)	0.17	0.65 (0.28–1.54)	0.33
**Medical History**						
T1DM	0.007	0.035	0.27 (0.05–1.47)	0.13	3.66 (0.65–6.23)	0.14
Anemia	0.001	0.026	1.19 (0.71–2.04)	0.5	0.85 (0.49–1.47)	0.55
Asthma	0.005	0.043	0.59 (0.30–1.14)	0.12	1.63 (0.83–3.23)	0.16
Kidney disease	0.001	0.015	0.40 (0.04-3.89)	0.43	2.82 (0.27–5.23)	0.38
Pregnancy	0.001	0.002	0.54 (0.06–4.84)	0.58	2.23 (0.23-4.23)	0.49
Smoking (cigarettes)	0.001	0.011	1.87 (0.39-8.97)	0.43	1.63 (0.33–8.04)	0.55
Smoking (shisha)	0.003	0.025	0.39 (0.07–2.01)	0.26	2.59 (0.48–7.61)	0.27

Note: Multivariate model adjusted for potential confounding variables such as age, marital status, and sleeping hours. Values significant at *p* < 0.05 level (bold).

**Table 6:** Association of food habits and reading food labels with coffee consumption.

A. Food Habits Associated with Coffee Consumption	Yes (820)
I. Adding sugar	
Yes	352 (42.9)
Sometimes	223 (27.2)
No	245 (29.9)
II. Adding artificial flavors (hazelnut, caramel, etc.)	
Yes	180 (22.0)
Sometimes	276 (33.6)
No	364 (44.4)
III. Adding sauces (chocolate, toffee, etc.)	
Yes	136 (16.6)
Sometimes	235 (28.7)
No	449 (54.7)
IV. Adding cream	
Yes	105 (12.8)
Sometimes	153 (18.7)
No	562 (68.5)
V. Adding spices (saffron, cardamom, etc.)	
Yes	590 (71.9)
Sometimes	57 (6.9)
No	173 (21.2)
VI. Drinking coffee immediately after meals	
Yes	207 (25.2)
Sometimes	32 (3.9)
No	581 (70.9)
VII. Drinking coffee before breakfast	
Yes	346 (42.2)
Sometimes	63 (7.7)
No	411 (50.1)
VIII. Increasing coffee intake during exams and school stress	
Yes	530 (64.6)
Sometimes	134 (16.4)
No	156 (19.0)
**B.** **Reading food labels associated with coffee consumption**	**Yes (820)**
I. Reading food label for coffee product	
Yes	109 (13.3)
Sometimes	367 (44.8)
No	344 (41.9)
II. Understanding contents of the food label	
Yes	200 (24.4)
Sometimes	277 (33.8)
No	343 (41.8)
III. Food label affects coffee purchasing choice	
Yes	173 (21.1)
Sometimes	217 (26.5)
No	430 (52.4)
IV. Ingredients of the coffee from coffee shops	
Yes	188 (22.9)
Sometimes	195 (23.8)
No	437 (53.3)

Note: Data are presented as *N* (%) for categorical variables.
